# The association between insulin resistance-related markers and ASCVD with hyperuricemia: results from the 2005 to 2018 National Health and Nutrition Examination Survey

**DOI:** 10.3389/fcvm.2025.1583944

**Published:** 2025-05-23

**Authors:** Yujie Shi, Zuo Zhang, Jiajun Yang, Song Cui

**Affiliations:** Beijing Anzhen Hospital, Capital Medical University, Beijing, China

**Keywords:** insulin resistance, ASCVD, hyperuricemia, NHANES, TyG

## Abstract

**Background:**

The association between insulin resistance and the comorbidity of ASCVD with hyperuricemia remains to be further explored. This study utilizes several insulin resistance indicators, including HOMA-IR, METS-IR, TyG, TyG-WHtR, TyG-WC, and TyG-BMI, to assess insulin resistance levels and investigate their association with the comorbidity of ASCVD and hyperuricemia in the study population.

**Methods:**

This cross-sectional study included 16,092 participants from the National Health and Nutrition Examination Survey conducted between 2005 and 2018. Participants younger than 20 years old and those with missing data for exposure-related indicators were excluded. Six insulin resistance-related traditional and novel indicators—HOMA-IR, METS-IR, TyG, TyG-WC, TyG-WHtR, and TyG-BMI—were used as exposure factors, while the outcome was ASCVD with hyperuricemia. This study's analyses incorporated sample weights, clustering, and stratification to account for the complex multi-stage stratified probability sampling design employed in NHANES.

**Results:**

A total of 523 patients were diagnosed with ASCVD with hyperuricemia. The results were adjusted for different covariates. METS-IR showed a consistent positive correlation with the outcome in all models, with model 3 indicating that for each unit increase, the OR was 1.04 (95% CI: 1.03–1.05, *p* < 0.0001). The study results showed that TyG-WC, TyG-WHtR, and TyG-BMI remained significantly associated across all models, with TyG-WHtR exhibiting the strongest association (OR = 1.64, 95% CI: 1.37–1.97, *p* < 0.0001). Furthermore, RCS results showed significant nonlinear relationships for HOMA-IR, METS-IR, TyG-WC, TyG-WHtR, and TyG-BMI with ASCVD with hyperuricemia (*p*-overall < 0.05, *p*-nonlinear < 0.05). The ROC analysis revealed high AUC values for TyG-BMI and METS-IR, with AUCs of 0.942 and 0.941. TyG-WC and TyG-WHtR also showed relatively high AUC values of 0.902 and 0.899, respectively. In the calibration curve analysis, METS-IR demonstrated the highest calibration performance.

**Conclusions:**

This NHANES-based study highlighted significant associations between insulin resistance indices, particularly METS-IR, TyG-WC, and TyG-WHtR, and ASCVD with hyperuricemia. Furthermore, it demonstrated the strong predictive capabilities of these indices for identifying individuals at risk for this comorbidity. These findings offer valuable insights into early detection and preventive strategies for ASCVD combined with hyperuricemia, emphasizing the practicality of these indices in clinical and public health settings.

## Introduction

Atherosclerotic cardiovascular disease (ASCVD), including coronary heart disease (CHD), angina/angina pectoris, heart attack(HA), and stroke, is among the diseases with the highest incidence and mortality rates worldwide (1, 2). ASCVD and hyperuricemia often coexist, potentially forming a mutually reinforcing vicious cycle. For instance, individuals with hyperuricemia have a significantly increased risk of developing ASCVD, while patients with ASCVD frequently present with hyperuricemia (3). Hyperuricemia, as a metabolic disorder independent of metabolic syndrome, shares common pathological processes with ASCVD, including oxidative stress, chronic inflammatory responses, endothelial dysfunction, vascular smooth muscle cell proliferation, thrombogenic tendencies, and lipid metabolism disorders (4–6). These conditions often exacerbate each other, leading to their frequent co-occurrence. These shared pathological pathways may have contributed to the increasing prevalence of individuals with ASCVD combined with hyperuricemia. Meanwhile, common dietary habits and lifestyle factors, such as high intake of purine-rich foods, excessive sugar consumption, obesity, and sedentary behavior, might act as shared upstream drivers of both conditions (3, 7). Consequently, research focused on this population is receiving increasing attention.

The relationship between insulin resistance (IR) and ASCVD has been well-documented. Existing studies and meta-analyses indicate that IR is significantly associated with ASCVD and serves as an effective predictor for ASCVD, showing IR as a major risk factor contributing to ASCVD through various metabolic pathways (8–12). Pino et al. identified three etiological pathways linking IR to ASCVD: the fundamental molecular mechanisms of insulin resistance, compensatory hyperinsulinemia that occurs following insulin resistance, and the association between insulin resistance and a series of cardiac metabolic abnormalities (13). IR induces several pathological mechanisms that lead to vascular damage, such as inflammation, oxidative stress, and lipid metabolism alterations, all of which play critical roles in plaque formation and arterial stiffness (14). Key IR markers, like the triglyceride-glucose (TyG) index and Homeostasis Model Assessment of Insulin Resistance (HOMA-IR), are widely studied for their predictive value in assessing ASCVD risk (13). Meta-analyses have reinforced the significance of these markers, suggesting their potential utility in early ASCVD risk stratification in both diabetic and non-diabetic populations (15). Insulin resistance and hyperuricemia are closely linked physiologically. Insulin resistance can increase uric acid production and reduce renal excretion, resulting in elevated uric acid levels (16). Additionally, uric acid itself inhibits insulin signaling in endothelial cells, exacerbating insulin resistance and creating a vicious cycle (5, 16, 17). Shared pathological processes, including oxidative stress and inflammatory responses, may mutually amplify these metabolic disorders, thereby escalating cardiovascular risk. However, to date, the relationship between insulin resistance and the specific disease state of ASCVD combined with hyperuricemia remains under-researched and unconfirmed.

In this study, we focused on ASCVD with hyperuricemia to investigate the association between insulin resistance-related markers, including HOMA-IR, METS-IR, TyG, TyG-WC, TyG-WHtR, and TyG-BMI, and this comorbid condition. We evaluated the predictive capabilities and clinical utility of each marker to provide scientific insights for the prevention and identification of this comorbidity in clinical settings.

## Methods

### Study design and participants

**Figure 1:**
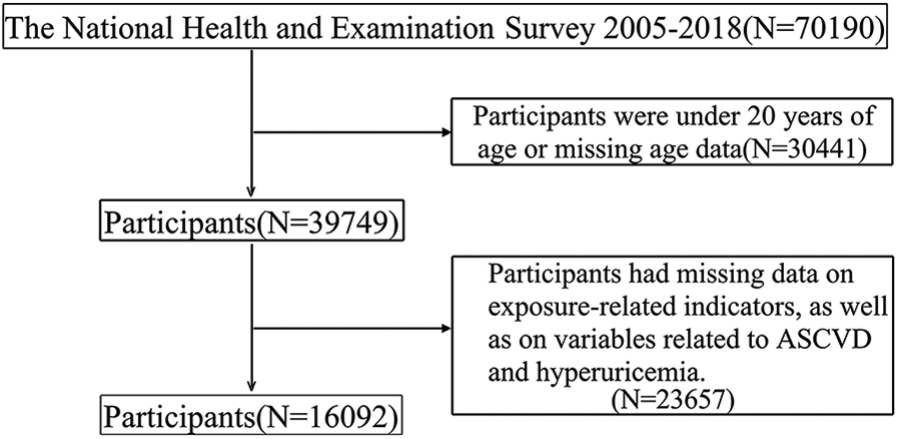
Flowchart of the study participants.

This is a cross-sectional study based on data from the National Health and Nutrition Examination Survey (NHANES) database covering the years 2005–2018. The NHANES is a large, continuous survey led by the National Center for Health Statistics (NCHS), aiming to assess the health and nutritional status of the U.S. population. All surveys conducted within NHANES have been approved by the NCHS Institutional Review Board, ensuring that informed consent is obtained from each participant. This study included 16,092 participants who took part in the NHANES survey from 2005 to 2018. We established exclusion criteria to screen the study population: (1) participants were under 20 years of age or missing age data; (2) participants had missing data for exposure-related indicators. Finally, a total of 16,092 participants were included in this study. The flowchart is presented in Figure 1.

### Definition of ASCVD with hyperuricemia

In this study, ASCVD was defined as self-reported presence of any of the following conditions: coronary heart disease, angina/angina pectoris, heart attack, or stroke. Participants were asked the following question: *Has a doctor or other health professional ever told {you/SP} that {you/s/he} had coronary heart disease, angina (also called angina pectoris), a heart attack (also called myocardial infarction), or a stroke?* A response of *Yes* was considered a positive self-report. The definition of hyperuricemia is a serum uric acid level of ≥7 mg/dl in men and ≥6 mg/dl in women. Individuals meeting the criteria for both ASCVD and hyperuricemia were defined as having ASCVD with hyperuricemia.

### Definition of HOMA-IR, METS-IR, TyG index, and TyG-related indexes

#### HOMA-IR

The HOMA-IR is a commonly used indicator for assessing insulin resistance, particularly in studies investigating the risk of metabolic syndrome, diabetes, and cardiovascular disease. It is calculated using fasting glucose and fasting insulin values, providing an estimate of insulin resistance through a simple formula. The calculation formula for HOMA-IR is as follows: $\mathrm{HOMA}-\mathrm{IR}=\frac{\mathrm{Fasting}\;\mathrm{Insulin}(\mu U/\mathrm{dL})}{405}$ (18).

#### METS-IR

The Metabolic Score for Insulin Resistance (METS-IR) is a metabolic score used to assess an individual's insulin resistance status. Unlike the traditional HOMA-IR, METS-IR does not require fasting insulin data, making it a convenient and broadly applicable indicator. METS-IR is calculated based on readily available measures such as blood glucose, lipid levels, and body weight, and can be used for risk assessment in conditions related to metabolic syndrome, including diabetes and cardiovascular disease. METS-IR is calculated using the following formula: $\mathrm{MET}-\mathrm{IR}=\frac{\ln\;((2\times \mathrm{FBG})+\mathrm{TG})\times \mathrm{BMI}}{\ln\;(\mathrm{HDL}-C)}$ (5).

#### TyG index

The Triglyceride-Glucose (TyG) Index is a metabolic marker based on triglyceride (TG) and fasting plasma glucose (FPG) levels in the blood, primarily used to assess individual IR. In recent years, the TyG Index has become an important tool in research and clinical settings for evaluating metabolic health, cardiovascular disease risk, and insulin resistance due to its simplicity, cost-effectiveness, and efficiency. The calculation formula is as follows: $\mathrm{TyG}=\frac{\ln\;(\mathrm{TG}(\mathrm{mg}/\mathrm{dL})\times \mathrm{FPG}(\mathrm{mg}/\mathrm{dL}))}{2}$ (19, 20).

#### TyG related index

TyG-WC, TyG-BMI, and TyG-WHtR are TyG-related indices used to assess insulin resistance and the risk of other diseases. These indices combine triglyceride and glucose levels with waist circumference, height, weight, and BMI, respectively, using innovative calculation methods to assess insulin resistance. The development of these indexes stems from the recognition that insulin resistance and visceral fat accumulation are major risk factors for various metabolic diseases. They are calculated as follows: (1) TyG−WC=TyG×WC(cm) (14); (2) TyG−BMI=TyG×BMI (14); (3) $\mathrm{WHtR}=\frac{\mathrm{Waist}\;\mathrm{Circumference}(\mathrm{cm})}{\mathrm{Height}(\mathrm{cm})}$; (4) TyG−WHtR=TyG×WHtR (14).

### Assessment of other variables

All covariates were treated as categorical variables. Participants were categorized by age into three groups: ≥20 and ≤35, >35 and ≤60, and >60. Educational attainment was classified as Less than high school, High school or equivalent, and College or above. Racial and ethnic were divided into five groups: Mexican American, other Hispanic, non-Hispanic white, non-Hispanic black, and other race. Marital status was grouped into two categories: Married or living with partner, and Widowed, Divorced, or Separated. Poverty-to-Income Ratio (PIR) was categorized as <1, ≥ 1 and <3, and ≥3. Smoking status was classified as never, former, or current, based on participants' smoking history and current smoking status. Alcohol consumption was categorized into two groups: yes and no. BMI was classified as follows: underweight (<18.5), normal weight (18.5 ≤ BMI < 25), overweight (25 ≤ BMI < 30), and obese (BMI ≥ 30). Hypertension was defined as meeting any of the following criteria: (1) systolic blood pressure (SBP) ≥ 140 mmHg; (2) diastolic blood pressure (DBP) ≥ 80 mmHg; (3) self-reported diagnosis of hypertension. Meeting any of the following criteria was defined as diabetes: (1) FBG ≥ 126 mg/dl; (2) glycated hemoglobin (HbA1c) ≥ 6.5%; (3) self-reported diagnosis of diabetes. Hyperlipidemia was defined by any of the following criteria: (1) LDL cholesterol ≥ 160 mg/dl; (2) total cholesterol ≥ 240 mg/dl; (3) current use of lipid-lowering medications. Further details regarding the covariates are available on the NHANES website (https://www.cdc.gov/nchs/nhanes/index.htm).

### Statistical analysis

The analyses for this study accounted for sample weights, clustering, and stratification to accommodate the complex multi-stage stratified probability sampling design used in NHANES. All statistical analyses were conducted following CDC guidelines. Continuous variables were described using mean and standard error, while categorical variables were summarized using frequency and percentages. In this study, HOMA-IR, TyG index, TyG-WC, TyG-BMI, and TyG-WHtR were initially analyzed as continuous variables. Subsequently, these variables were categorized into quartiles (Q1, Q2, Q3, Q4) for further analysis, with Q1 serving as the reference group. A two-sided *p*-value of less than 0.05 was considered statistically significant. Baseline characteristics of the population were described based on the presence or absence of ASCVD with hyperuricemia. Weighted restricted cubic spline (RCS) analysis was applied to explore the dose-response relationship between insulin resistance-related indices and ASCVD with hyperuricemia. All confounding factors were adjusted for in the RCS analysis model. The data analysis was conducted using R version 4.4.1.

## Results

### Baseline characteristics

Participants were categorized and descriptively analyzed based on the presence of ASCVD with hyperuricemia, with baseline characteristics summarized in Table 1. Compared with other groups, individuals with ASCVD combined with hyperuricemia were characterized by older age, higher BMI, central obesity, and a higher prevalence of hypertension and diabetes.

**Table 1:** Baseline.

Characteristics	ALL	ASCVD with hyperuricemia	ASCVD without hyperuricemia	Non-ASCVD with hyperuricemia	Non-ASCVD without hyperuricemia	*p*.overall
	*N* = 16,092	*N* = 523	*N* = 1,057	*N* = 2,835	*N* = 11,677	
Age						0.000
>20,≤35	4,317 (26.8%)	7 (1.34%)	25 (2.37%)	653 (23.0%)	3,632 (31.1%)	
>35,≤60	6,829 (42.4%)	123 (23.5%)	298 (28.2%)	1,166 (41.1%)	5,242 (44.9%)	
>60	4,946 (30.7%)	393 (75.1%)	734 (69.4%)	1,016 (35.8%)	2,803 (24.0%)	
Gender						<0.001
Male	7,818 (48.6%)	283 (54.1%)	626 (59.2%)	1,536 (54.2%)	5,373 (46.0%)	
Female	8,274 (51.4%)	240 (45.9%)	431 (40.8%)	1,299 (45.8%)	6,304 (54.0%)	
Education						<0.001
Less than high school	4,025 (25.0%)	168 (32.2%)	367 (34.7%)	626 (22.1%)	2,864 (24.5%)	
High school or equivalent	8,311 (51.7%)	285 (54.6%)	504 (47.7%)	1,553 (54.8%)	5,969 (51.2%)	
College or above	3,742 (23.3%)	69 (13.2%)	186 (17.6%)	653 (23.1%)	2,834 (24.3%)	
Race						<0.001
Mexican American	2,566 (15.9%)	41 (7.84%)	111 (10.5%)	329 (11.6%)	2,085 (17.9%)	
Other Hispanic	1,624 (10.1%)	37 (7.07%)	98 (9.27%)	232 (8.18%)	1,257 (10.8%)	
Non-Hispanic White	6,848 (42.6%)	286 (54.7%)	573 (54.2%)	1,223 (43.1%)	4,766 (40.8%)	
Non-Hispanic Black	3,241 (20.1%)	131 (25.0%)	196 (18.5%)	687 (24.2%)	2,227 (19.1%)	
Other race	1,813 (11.3%)	28 (5.35%)	79 (7.47%)	364 (12.8%)	1,342 (11.5%)	
Marital						<0.001
Married/Living with partner	9,821 (61.0%)	286 (54.7%)	622 (58.8%)	1,683 (59.4%)	7,230 (61.9%)	
Widowed/divorced/separated/never married	6,267 (39.0%)	237 (45.3%)	435 (41.2%)	1,152 (40.6%)	4,443 (38.1%)	
PIR						<0.001
<1	4,418 (27.5%)	160 (30.6%)	303 (28.7%)	704 (24.8%)	3,251 (27.8%)	
≥1,<3	6,222 (38.7%)	239 (45.7%)	482 (45.6%)	1,109 (39.1%)	4,392 (37.6%)	
≥3	5,452 (33.9%)	124 (23.7%)	272 (25.7%)	1,022 (36.0%)	4,034 (34.5%)	
Alcohol						<0.001
No	5,930 (36.9%)	267 (51.1%)	515 (48.7%)	980 (34.6%)	4,168 (35.7%)	
Yes	10,162 (63.1%)	256 (48.9%)	542 (51.3%)	1,855 (65.4%)	7,509 (64.3%)	
Smoke						<0.001
Never	8,848 (55.0%)	217 (41.5%)	387 (36.6%)	1,517 (53.5%)	6,727 (57.7%)	
Former	3,955 (24.6%)	214 (40.9%)	401 (37.9%)	791 (27.9%)	2,549 (21.9%)	
Current	3,275 (20.4%)	92 (17.6%)	269 (25.4%)	525 (18.5%)	2,389 (20.5%)	
BMI						<0.001
Underweight	258 (1.60%)	4 (0.76%)	17 (1.61%)	8 (0.28%)	229 (1.96%)	
Normal weight	4,454 (27.7%)	65 (12.4%)	259 (24.5%)	390 (13.8%)	3,740 (32.0%)	
Overweight	5,377 (33.4%)	152 (29.1%)	369 (34.9%)	855 (30.2%)	4,001 (34.3%)	
Obese	6,003 (37.3%)	302 (57.7%)	412 (39.0%)	1,582 (55.8%)	3,707 (31.7%)	
WC						<0.001
Central obesity	13,817 (85.9%)	506 (96.7%)	989 (93.6%)	2,692 (95.0%)	9,630 (82.5%)	
Normal	2,275 (14.1%)	17 (3.25%)	68 (6.43%)	143 (5.04%)	2,047 (17.5%)	
Hypertension						<0.001
Yes	6,813 (42.3%)	449 (85.9%)	788 (74.6%)	1,635 (57.7%)	3,941 (33.8%)	
No	9,279 (57.7%)	74 (14.1%)	269 (25.4%)	1,200 (42.3%)	7,736 (66.2%)	
Diabetes						<0.001
Yes	2,940 (18.3%)	253 (48.4%)	408 (38.6%)	650 (22.9%)	1,629 (14.0%)	
No	13,152 (81.7%)	270 (51.6%)	649 (61.4%)	2,185 (77.1%)	10,048 (86.0%)	
Hyperlipidemia						<0.001
Yes	5,026 (31.2%)	316 (60.4%)	645 (61.0%)	1,026 (36.2%)	3,039 (26.0%)	
No	11,066 (68.8%)	207 (39.6%)	412 (39.0%)	1,809 (63.8%)	8,638 (74.0%)	

### Relationship between HOMA-IR, METS-IR, TyG index, TyG-related indexes and ASCVD with hyperuricemia

The results showed that after adjusting for covariates. Three logistic regression models were constructed for this analysis: (1) model 1: an unadjusted model; (2) model 2: adjusted for Age, Gender, Education, Race, Marital Status, and PIR; (3) model 3: further adjusted based on model 2 by including Alcohol, Smoking, BMI, WC, Hypertension, Diabetes, and Hyperlipidemia. These three models were used to evaluate the odds ratios (ORs) and 95% confidence intervals (CIs) for the association between different insulin resistance-related indices and ASCVD with hyperuricemia among participants. The *F*-test was used to assess the goodness of fit for the model. HOMA-IR was significantly associated with the ASCVD with hyperuricemia in model 1 (OR = 1.03, 95% CI: 1.01–1.04, *p* < 0.0001). The association persisted in Model 2 but was attenuated in Model 3, where only Quartile 4 retained significance (OR = 1.92, 95% CI: 1.14–3.22, *p* = 0.015). METS-IR showed a consistent positive association with the outcome across all models, which Model 3 revealing an OR of 1.04 per unit increase (95% CI: 1.03–1.05, *p* < 0.0001), with Quartile 4 showing an OR of 4.48 (2.00–10.03, *p* = 0.0004). TyG demonstrated a significant association with ASCVD with hyperuricemia in Models 1 and 2. However, after adjusting for a more comprehensive set of confounders in Model 3, the strength of these associations diminished and lost statistical significance. TyG-WC, TyG-WHtR, and TyG-BMI remained significantly associatied across all models, with TyG-WHtR showing the most robust association (OR = 1.64, 95% CI: 1.37–1.97, *p* < 0.0001). Quartile 4 of TyG-WHtR exhibited the strongest association (OR = 6.17 95% CI: 2.56–14.91, *p* < 0.0001), followed by TyG-WC (Q4: OR = 6.17, 95% CI: 2.56–14.91, *p* < 0.0001). The analysis demonstrates that higher levels of METS-IR, TyG-WC, and TyG-WHtR are consistently associated with increased risk across models, with the strongest effects observed in the highest quartiles. The results are presented in Table 2. The same analysis was also conducted in populations with ASCVD without hyperuricemia, non-ASCVD with hyperuricemia, and those with neither condition. The results are presented in Supplementary Table S1. The results indicate that, in the association between insulin resistance and ASCVD with concomitant hyperuricemia, ASCVD carries greater relative weight.

**Table 2:** The relationship between IR-related indexes with ASCVD with hyperuricemia.

Variables	Model 1			Model 2			Model 3		
	OR	95%CI	*p*-value	OR	95%CI	*p*-value	OR	95%CI	*p*-value
HOMA-IR	1.03	1.01,1.04	<0.0001	1.02	1.01, 1.03	<0.0001	1.01	0.99, 1.02	0.09
As categorical variables (quartile)									
Q1	1 (reference)			1 (reference)			1 (reference)		
Q2	1.47	0.98, 2.20	0.06	1.31	0.89, 1.91	0.17	1.11	0.72, 1.69	0.63
Q3	2.58	1.60, 4.15	0.0001	2.04	1.31, 3.18	0.002	1.38	0.83, 2.28	0.21
Q4	5.23	3.34, 8.20	<0.0001	3.90	2.58, 5.91	<0.0001	1.92	1.14, 3.22	0.015
METS-IR	1.04	1.03, 1.05	<0.0001	1.05	1.04, 1.06	<0.0001	1.04	1.03, 1.05	<0.0001
As categorical variables (quartile)									
Q1	1 (reference)			1 (reference)			1 (reference)		
Q2	1.86	1.01, 3.40	0.04	1.41	0.79, 2.53	0.24	1.62	0.82, 3.20	0.16
Q3	2.88	1.67, 4.99	0.0002	2.24	1.32, 3.81	0.003	2.54	1.16, 5.57	0.02
Q4	5.31	3.06, 9.22	<0.0001	4.47	2.64, 7.55	<0.0001	4.48	2.00, 10.03	0.0004
TyG	2.12	1.81, 2.48	<0.0001	1.90	1.56, 2.31	<0.0001	1.23	0.98, 1.56	0.08
As categorical variables (quartile)									
Q1	1 (reference)			1 (reference)			1 (reference)		
Q2	1.66	0.98, 2.82	0.06	1.21	0.70, 2.08	0.49	0.94	0.53, 1.67	0.83
Q3	2.96	1.72, 5.08	0.0001	1.98	1.13, 3.47	0.02	1.29	0.70, 2.39	0.41
Q4	5.17	3.02, 8.85	<0.0001	3.23	1.87, 5.59	<0.0001	1.54	0.83, 2.87	0.17
TyG-WC	1.0038	1.0032, 1.0045	<0.0001	1.0036	1.0028, 1.0044	<0.0001	1.0026	1.0015, 1.0037	<0.0001
As categorical variables (quartile)									
Q1	1 (reference)			1 (reference)			1 (reference)		
Q2	3.09	1.50, 6.39	0.003	1.96	0.96, 3.98	0.06	2.32	0.96, 5.62	0.06
Q3	4.39	2.22, 8.67	<0.0001	2.51	1.29, 4.89	0.01	2.54	1.02, 6.33	0.05
Q4	9.87	4.96, 19.65	<0.0001	5.43	2.79, 10.54	<0.0001	4.05	1.55, 10.59	0.005
TyG-WHtR	1.99	1.80, 2.20	<0.0001	1.87	1.65, 2.14	<0.0001	1.64	1.37, 1.97	<0.0001
As categorical variables (quartile)									
Q1	1 (reference)			1 (reference)			1 (reference)		
Q2	3.57	1.84, 6.91	0.0002	2.08	1.09, 3.98	0.03	2.60	1.17, 5.77	0.02
Q3	5.82	2.97, 11.43	<0.0001	3.10	1.58, 6.05	0.002	3.70	1.65, 8.33	0.002
Q4	13.18	6.68, 26.00	<0.0001	6.62	3.42, 12.81	<0.0001	6.17	2.56, 14.91	<0.0001
TyG-BMI	1.007	1.006, 1.009	<0.0001	1.008	1.006, 1.01	<0.0001	1.006	1.003, 1.009	0.0001
As categorical variables (quartile)									
Q1	1 (reference)			1 (reference)			1 (reference)		
Q2	1.56	0.93, 2.61	0.09	1.08	0.65, 1.78	0.76	0.87	0.44, 1.72	0.68
Q3	2.62	1.61, 4.26	0.0002	1.87	1.16, 3.01	0.01	1.20	0.49, 2.98	0.68
Q4	4.38	2.65, 7.23	<0.0001	3.27	2.01, 5.31	<0.0001	1.51	0.54, 4.18	0.43

Logistic regression models were constructed for this analysis: (1)model 1: an unadjusted model; (2)model 2: adjusted for Age, Gender, Education, Race, Marital Status, and PIR; (3)model 3: further adjusted based on model 2 by including Alcohol, Smoking, BMI, WC, Hypertension, Diabetes, and Hyperlipidemia.

For CHD with hyperuricemia, HOMA-IR (OR = 1.01, 95% CI: 1.003–1.024, *p* = 0.01) shows a positive association in Q3 (OR = 2.55, 95% CI: 1.33–4.90, *p* = 0.005) and Q4 (OR = 3.70, 95% CI: 1.80–7.61, *p* = 0.0005). METS-IR (OR = 1.02, 95% CI: 1.005–1.04, *p* = 0.01) was significantly associaed in Q3 (OR = 7.81, 95% CI: 2.85–21.41, *p* = 0.0001) and Q4 (OR = 9.86, 95% CI: 3.16–30.72, *p* = 0.0001). TyG-WHtR (OR = 1.43, 95% CI: 1.15–1.77, *p* = 0.001) was found significantly associated with CHD with hyperuricemia in Q2, Q3, and Q4, with the highest risk in Q4 (OR = 13.88, 95% CI: 3.64–52.94, *p* = 0.0002). For angina with hyperuricemia, positive association across Q2, Q3, and Q4 were observed in METS-IR (OR = 1.03, 95% CI: 1.01–1.05, *p* = 0.001), TyG (OR = 1.48, 95% CI: 1.11–1.96, *p* = 0.008) and TyG-WC (OR = 1.002, 95% CI: 1.0004–1.003, *p* = 0.009), with Q4 showing the strongest effect (METS-IR: OR = 15.66, 95% CI: 4.08–60.10, *p* = 0.0001; TyG: OR = 5.82, 95% CI: 2.01–16.87, *p* = 0.001; TyG-WC: OR = 18.90, 95% CI: 4.56–78.42, *p* < 0.0001). TyG-WHtR showed significant associations across all quartiles, with Q4 having the highest OR of 9.46 (95% CI: 1.82–49.24, *p* = 0.008). For heart attack with hyperuricemia, METS-IR (OR = 1.04, 95% CI: 1.02–1.06, *p* = 0.0006), TyG-WHtR (OR = 1.62, 95% CI: 1.27–2.05, *p* = 0.0001) and TyG-WC (OR = 1.002, 95% CI: 1.00041–1.004, *p* = 0.015) showed positive results. For each indicator, the Q4 level showed a significant association with heart attack with hyperuricemia (METS-IR: OR = 5.69, 95% CI: 1.78–18.23, *p* = 0.004; TyG-WHtR: OR = 39.93, 95% CI: 12.29–129.81, *p* < 0.0001; TyG-WC: OR = 5.56, 95% CI: 1.33–23.26, *p* = 0.02). For stroke with hyperuricemia, only TyG-WHtR (OR = 1.87, 95% CI: 1.39–2.52, *p* < 0.0001) and METS-IR (OR = 1.05, 95% CI: 1.03–1.07, *p* < 0.0001) showed significant results. The results are presented in Table 3.

**Table 3:** The relationship between IR-related indexes with CHD, angina, heart attack, and stroke with hyperuricemia.

Variables	HOMA-IR			METS-IR			TyG			TyG-WC			TyG-WHtR			TyG-BMI		
	OR	95% CI	*p*-value	OR	95% CI	*p*-value	OR	95% CI	*p*-value	OR	95% CI	*p*-value	OR	95% CI	*p*-value	OR	95% CI	*p*-value
CHD with hyperuricemia	1.01	1.003,1.024	0.01	1.02	1.005, 1.04	0.01	1.27	0.92, 1.74	0.14	1.002	1.0004, 1.003	0.01	1.43	1.15, 1.77	0.001	1.002	0.99, 1.005	0.24
As categorical variables (quartile)																		
Q1	1 (reference)			1 (reference)			1 (reference)			1 (reference)			1 (reference)			1 (reference)		
Q2	1.37	0.68, 2.76	0.38	2.51	0.99, 6.37	0.05	1.19	0.45, 3.12	0.72	5.13	1.32, 19.96	0.02	5.43	1.64,17.93	0.006	1.34	0.51, 3.57	0.55
Q3	2.55	1.33, 4.90	0.005	7.81	2.85, 21.41	0.0001	1.51	0.56, 4.07	0.41	5.97	1.57, 22.70	0.009	8.88	2.72, 28.92	0.04	3.76	1.22, 11.55	0.02
Q4	3.70	1.80, 7.61	0.0005	9.86	3.16, 30.72	0.0001	1.98	0.77, 5.10	0.15	8.74	2.06, 37.19	0.004	9.46	1.82, 49.24	0.008	2.94	0.82, 10.48	0.1
Angina with hyperuricemia	1.007	0.998, 1.016	0.13	1.03	1.01, 1.05	0.001	1.48	1.11, 1.96	0.008	1.002	1.0004, 1.003	0.009	1.42	1.13, 1.79	0.003	1.004	1.0009, 1.008	0.015
As categorical variables (quartile)																		
Q1	1 (reference)			1 (reference)			1 (reference)			1 (reference)			1 (reference)			1 (reference)		
Q2	3.17	1.43, 7.04	0.005	4.15	1.35, 12.73	0.01	2.24	0.83, 6.08	0.11	12.18	2.89, 51.38	0.0008	4.47	1.02, 19.49	0.05	1.85	0.51, 6.74	0.35
Q3	3.81	1.51, 9.64	0.005	8.52	2.54, 28.62	0.0007	4.25	1.45, 12.48	0.009	18.22	4.49, 73.86	<0.0001	5.38	1.12, 25.86	0.04	4.64	1.20, 17.95	0.03
Q4	6.81	2.56, 18.13	0.0002	15.66	4.08, 60.10	0.0001	5.82	2.01, 16.87	0.001	18.90	4.56, 78.42	<0.0001	9.46	1.82, 49.24	0.008	5.09	1.20, 21.54	0.03
Heart attack with hyperuricemia	0.997	0.98, 1.01	0.66	1.04	1.02, 1.06	0.0006	1.13	0.81, 1.59	0.47	1.002	1.0004, 1.004	0.015	1.62	1.27, 2.05	0.0001	1.004	0.99, 1.008	0.06
As categorical variables (quartile)																		
Q1	1 (reference)			1 (reference)			1 (reference)			1 (reference)			1 (reference)			1 (reference)		
Q2	1.28	0.56, 2.93	0.55	1.87	0.71, 4.93	0.20	1.64	0.63, 4.28	0.31	3.31	1.06, 10.39	0.04	14.71	5.56, 38.89	<0.0001	0.53	0.18, 1.58	0.25
Q3	1.64	0.65, 4.12	0.29	2.91	0.95, 8.95	0.06	1.32	0.45, 3.84	0.61	3.04	0.90, 10.25	0.07	19.58	7.04, 54.46	<0.0001	0.52	0.14, 1.94	0.32
Q4	2.10	0.79, 5.53	0.13	5.69	1.78, 18.23	0.004	1.55	0.51, 4.71	0.44	5.56	1.33, 23.26	0.02	39.93	12.29, 129.81	<0.0001	0.66	0.14, 2.98	0.58
Stroke with hyperuricemia	1.002	0.98, 1.02	0.86	1.05	1.03, 1.07	<0.0001	1.27	0.90, 1.79	0.16	1.004	1.002, 1.006	<0.0001	1.87	1.39, 2.52	<0.0001	1.009	1.005, 1.013	<0.0001
As categorical variables (quartile)																		
Q1	1 (reference)			1 (reference)			1 (reference)			1 (reference)			1 (reference)			1 (reference)		
Q2	0.85	0.47, 1.54	0.6	2.07	0.77, 5.56	0.15	1.00	0.52, 1.91	0.99	1.02	0.39, 2.68	0.96	0.97	0.31, 3.07	0.96	1.35	0.54, 3.39	0.52
Q3	0.93	0.50, 1.71	0.8	0.93	0.30, 2.82	0.89	1.17	0.57, 2.37	0.67	1.20	0.40, 3.62	0.74	1.36	0.39, 4.74	0.63	0.87	0.26, 2.95	0.83
Q4	0.98	0.55, 1.74	0.93	2.85	0.90, 9.05	0.08	1.25	0.64, 2.41	0.51	1.93	0.66, 5.60	0.23	2.31	0.64, 8.42	0.20	1.76	0.48, 6.44	0.39

### Restricted cubic splines analysis

**Figure 2:**
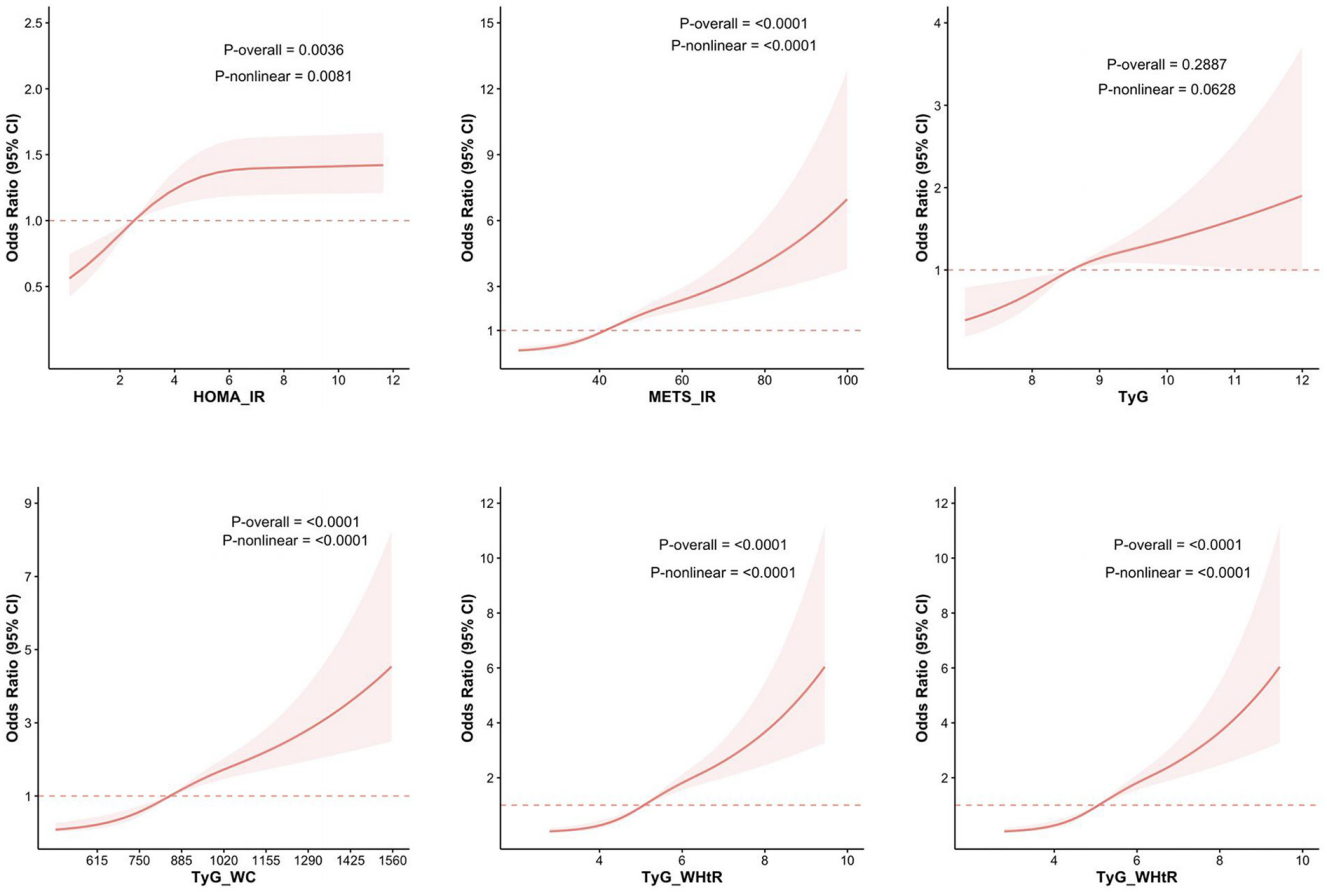
RCS analysis of the relationship between IR-related indexes and the risk of ASCVD with hyperuricemia. The figure presents the results of RCS analysis examining the associations between IR-related indices—HOMA-IR, METS-IR, TyG, TyG-WC, TyG-WHtR, and TyG-BMI—and the risk of ASCVD with hyperuricemia.

Restricted cubic spline was utilized to better evaluate and illustrate the associations between HOMA-IR, METS-IR, TyG, TyG-WC, TyG-WHtR, TyG-BMI, and ASCVD with hyperuricemia. The results, depicted in Figure 2, reveal significant nonlinear relationships for HOMA-IR, METS-IR, TyG-WC, TyG-WHtR, and TyG-BMI with ASCVD with hyperuricemia (*p*-overall < 0.05, *P*-nonlinear < 0.05).

**Figure 3:**
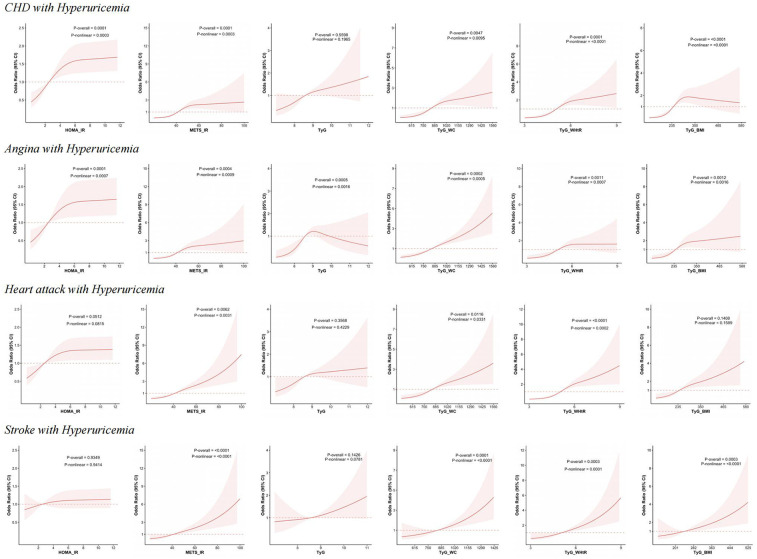
RCS analysis of IR-related indexes with the risk of CHD, angina, heart attack and stroke with hyperuricemia. This figure presents the results of RCS analysis examining the relationship between HOMA-IR, METS-IR, TyG, TyG-WC, TyG-WHtR, and TyG-BMI with the risk of CHD, angina, heart attack and stroke with hyperuricemia.

Similarly, nonlinear associations were observed between the same five indices and CHD combined with hyperuricemia. Futhermore, all six indices, including HOMA-IR, METS-IR, TyG, TyG-WC, TyG-WHtR, and TyG-BMI, demonstrate nonlinear associations with angina with hyperuricemia. For heart attack combined with hyperuricemia, only METS-IR, TyG-WC, and TyG-WHtR exhibit nonlinear associations. In the case of stroke with hyperuricemia, METS-IR, TyG-WC, TyG-WHtR, and TyG-BMI exhibit nonlinear associations. Figure 3 illustrates the above results.

### Receiver operating characteristics curves

**Figure 4:**
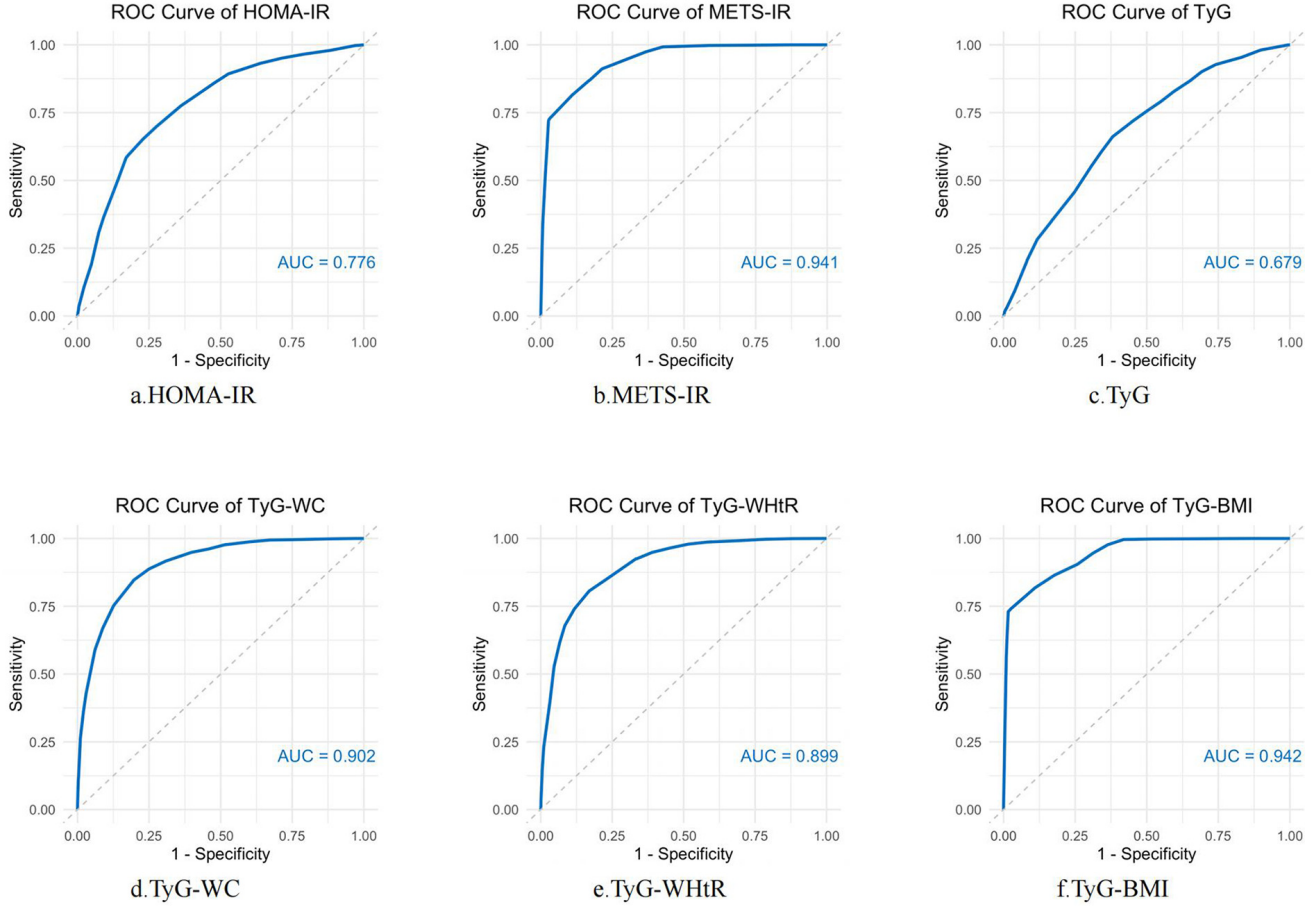
ROC curve analysis of IR-related indexes in predicting ASCVD with hyperuricemia. ROC curves were constructed to evaluate the performance of several insulin resistance markers—HOMA-IR, METS-IR, TyG, TyG-WC, TyG-WHtR, and TyG-BMI—in predicting the occurrence of ASCVD with hyperuricemia. AUC was used to assess the diagnostic accuracy of each marker. Higher AUC values indicate better predictive power.

Figure 4 illustrates the receiver operating characteristics (ROC) curves of different indicators (HOMA-IR, METS-IR, TyG, TyG-WC, TyG-WHtR, TyG-BMI) in predicting ASCVD with hyperuricemia. In predicting ASCVD with hyperuricemia, the AUC values of the indicators ranged from 0.679 to 0.942, demonstrating similar predictive capabilities. Among the indices, TyG-BMI had the highest AUC value (AUC = 0.942), closely followed by METS-IR (AUC = 0.941). Both TyG-WC and TyG-WHtR also demonstrate good performance, with AUC values of 0.902 and 0.899, respectively. Relatively speaking, the predictive ability of HOMA-IR (AUC = 0.776) and TyG (AUC = 0.679) is somewhat lower.

### Subgroup analysis

The results of the subgroup analysis are presented in Table 4. The results of the subgroup analysis showed that METS-IR, TyG-WC, and TyG-WHtR demonstrated positive associations with ASCVD with hyperuricemia across almost all subgroups. HOMA-IR showed significant results in subgroups such as individuals aged over 60, females, those with an education level of less than high school, PIR < 1, widowed/divorced/separated/never married individuals, non-smokers, those with normal weight, non-central obesity, individuals with hypertension, and those without diabetes. TyG exhibited notable significance in groups with less than high school or high school equivalent education levels, PIR ≥ 1 and < 3, current smokers, those with normal BMI, non-central obesity, and individuals without diabetes or hyperlipidemia. TyG-BMI performed well across all age groups, males, individuals with less than high school or high school equivalent education levels, PIR < 3, irrespective of marital status or alcohol consumption, former and current smokers, individuals with normal BMI or obesity, regardless of central obesity status, and those with or without hypertension, diabetes, and hyperlipidemia. This underscores the versatility and reliability of TyG-BMI across diverse demographic and clinical characteristics.

**Table 4:** Subgroup analysis.

Subgroup	No. ASCVD with hyperuricemia/total	HOMA-IR	METS-IR	TyG	TyG-WC	TyG-WHTR	TyG-BMI
		OR (95%C I) *p*-value	OR (95% CI) *p*-value	OR (95% CI) *p*-value	OR (95% CI) *p*-value	OR (95% CI) *p*-value	OR (95% CI) *p*-value
Age (years)							
>20, ≤35	7/4,324	1,02 (0.99, 1.05) 0.17	1.06 (1.01, 1.11) 0.02	1.70 (0.46, 6.35) 0.42	1.005 (1.00, 1.01) 0.08	2.48 (0.79, 7.80) 0.12	1.01 (1.00, 1.03)0.05
>35, ≤60	123/6,857	1.00 (0.99, 1.01) 0.73	1.04 (1.02, 1.06) < 0.0001	1.27 (0.88, 1.84) 0.20	1.003 (1.002, 1.005) 0.0002	1.78 (1.37, 2.31) < 0.0001	1.006 (1.00, 1.01) 0.003
>60	393/4,985	1.01 (1.00, 1.02) 0.08	1.03 (1.02, 1.05) < 0.0001	1.16 (0.85, 1.59) 0.33	1.002 (1.0007,1.004) 0.004	1.54 (1.22, 1.95) 0.0004	1.004 (1.0008, 1.008) 0.016
Gender							
Male	283/7,858	0.999 (0.987, 1.011) 0.87	1.05 (1.03, 1.07) < 0.0001	1.21 (0.89, 1.66) 0.23	1.003 (1.002, 1.005) < 0.0001	2.03 (1.54, 2.66) < 0.0001	1.008 (1.004, 1.012) 0.002
Female	240/8,308	1.02 (1.01, 1.03) 0.004	1.03 (1.01, 1.05) 0.01	1.21 (0.84, 1.74) 0.31	1.002 (1.0003, 1.003) 0.02	1.37 (1.06, 1.76) 0.015	1.004 (1.00, 1.008) 0.07
Education							
Less than high school	168/4,051	1.01 (1.00, 1.02) 0.03	1.03 (1.01, 1.05) 0.01	1.48 (1.09, 2.02) 0.01	1.002 (1.001, 1.004) 0.005	1.65 (1.23, 2.23) 0.001	1.005 (1.00, 1.01) 0.05
High school or equivalent	285/8,352	1.01 (1.00, 1.02) 0.24	1.05 (1.03, 1.07) < 0.0001	1.40 (1.12, 1.76) 0.004	1.003 (1.002, 1.005) < 0.0001	1.72 (1.38, 2.13) < 0.0001	1.007 (1.003, 1.01) 0.0001
College or above	69/3,749	1.01 (0.99, 1.04) 0.29	1.02 (0.98, 1.06) 0.25	0.67 (0.27, 1.67) 0.38	1.001 (0.997, 1.005) 0.57	1.46 (0.83, 2.58) 0.19	1.002 (0.994, 1.011) 0.58
PIR							
<1	160/4,453	1.01 (1.00, 1.03) 0.06	1.04 (1.02, 1.06) 0.001	1.25 (0.82, 1.90) 0.30	1.003 (1.0005, 1.005) 0.02	1.72 (1.24, 2.39) 0.001	1.006 (1.001,1.01) 0.01
≥1,<3	239/6,251	1.00 (0.98, 1.01) 0.68	1.04 (1.02, 1.06) < 0.0001	1.46 (1.10, 1.94) 0.01	1.003 (1.001, 1.004) 0.0002	1.70 (1.29, 2.24) 0.0003	1.006 (1.002, 1.01) 0.03
≥3	124/5,462	1.00 (0.99,1.02) 0.64	1.03 (1.01, 1.05) 0.008	1.01 (0.59, 1.74) 0.97	1.002 (1.00, 1.004) 0.04	1.47 (1.05, 2.07) 0.03	1.003 (0.999, 1.008) 0.17
Marital status							
Married/Living with partner	286/9,860	1.00 (0.99, 1.01) 0.68	1.04 (1.02, 1.06) < 0.0001	1.23 (0.91, 1.66) 0.17	1.003 (1.001, 1.005) 0.0002	1.77 (1.38, 2.28) < 0.0001	1.007 (1.003, 1.01) 0.001
Widowed/Divorced/Separated/Never married	237/6,301	1.02 (1.01, 1.03) 0.0007	1.04 (1.02, 1.06) 0.0004	1.21 (0.80, 1.82)0.36	1.002 (1.0004, 1.004) 0.02	1.47 (1.10, 1.95) 0.009	1.004 (1.0001, 1.008) 0.04
Alcohol							
Yes	256/10,198	1.01 (1.00, 1.02) 0.1	1.04 (1.02, 1.06) < 0.0001	1.25 (0.89, 1.75) 0.19	1.002 (1.001, 1.004) 0.001	1.63 (1.27, 2.10) 0.0002	1.006 (1.001, 1.01) 0.01
No	267/5,968	1.01 (0.99, 1.02) 0.35	1.04 (1.02, 1.06) < 0.0001	1.20 (0.93, 1.55) 0.16	1.003 (1.001, 1.004) 0.0004	1.66 (1.32, 2.10) < 0.0001	1.005 (1.002, 1.009) 0.004
Smoke							
Current	92/3,296	1.01 (0.99, 1.04) 0.17	1.07 (1.04, 1.10) < 0.0001	1.47 (1.01, 2.15) 0.05	1.005 (1.003, 1.007) < 0.0001	2.52 (1.70, 3.74) < 0.0001	1.01 (1.007, 1.02) < 0.0001
Former	214/3,973	1.00 (0.98, 1.01) 0.48	1.04 (1.02, 1.07) 0.0003	1.08 (0.73, 1.59) 0.71	1.003 (1.001, 1.005) 0.002	1.70 (1.27, 2.28) 0.0005	1.006 (1.001, 1.01) 0.02
Never	217/8,883	1.01 (1.00, 1.03) 0.03	1.02 (1.00, 1.04) 0.05	1.28 (0.87, 1.88) 0.20	1.001 (1.00, 1.003) 0.16	1.33 (1.01, 1.74) 0.04	1.003 (0.999, 1.007) 0.18
BMI							
Normal weight	65/4,475	1.05 (1.01, 1.08) 0.01	1.13 (1.04, 1.22) 0.003	1.83 (0.92, 3.64) 0.08	1.006 (1.001, 1.01) 0.01	3.26 (1.48, 7.19) 0.004	1.02 (1.003, 1.04) 0.02
Overweight	152/5,399	1.02 (1.00, 1.04) 0.05	1.06 (1.02, 1.11) 0.006	1.34 (0.85, 2.12) 0.21	1.003 (1.00, 1.005) 0.05	1.99 (1.36, 2.91) 0.0006	1.009 (0.997, 1.02) 0.15
Obese	302/6,031	1.00 (0.99, 1.01) 0.66	1.04 (1.02, 1.05) < 0.0001	1.08 (0.78, 1.49) 0.65	1.003 (1.001, 1.004) < 0.0001	1.54 (1.25, 1.89) < 0.0001	1.006 (1.003, 1.008) 0.0003
WC							
Central obesity	506/13,882	1.01 (1.00, 1.02) 0.10	1.04 (1.02, 1.05) < 0.0001	1.19 (0.94, 1.51) 0.15	1.003 (1.001, 1.004) < 0.0001	1.61 (1.34, 1.94) < 0.0001	1.006 (1.003, 1.008) 0.0002
Normal	17/2,284	1.62 (0.97, 2.71) 0.06	1.11 (0.95, 1.30) 0.19	4.54 (1.03, 19.99) 0.05	1.02 (1.0005, 1.04) 0.05	18.39 (4.62, 73.16) < 0.0001	1.03 (1.004, 1.06) 0.03
Hypertension							
Yes	449/6,866	1.01 (1.00, 1.02) 0.03	1.04 (1.02, 1.05) < 0.0001	1.23 (0.95, 1.59) 0.11	1.002 (1.001, 1.003) < 0.0001	1.56 (1.32, 1.86) < 0.0001	1.006 (1.003, 1.008) 0.0002
No	74/9,300	0.99 (0.96, 1.01) 0.25	1.05 (1.02, 1.08) 0.001	1.30 (0.77, 2.19) 0.33	1.004 (1.002, 1.007) 0.001	2.18 (1.41, 3.38) 0.0006	1.007 (1.0009, 1.01) 0.03
Diabetes							
Yes	253/2,961	1.00 (0.99, 1.02) 0.36	1.04 (1.02, 1.06) < 0.0001	1.11 (0.85, 1.45) 0.44	1.003 (1.001, 1.004) 0.0008	1.64 (1.30, 2.07) < 0.0001	1.006 (1.003, 1.01) 0.001
No	270/13,205	1.07 (1.02, 1.11) 0.002	1.04 (1.02, 1.06) 0.0002	1.38 (0.95, 2.01) 0.09	1.003 (1.001, 1.004) 0.002	1.64 (1.25, 2.16) 0.0005	1.005 (1.0006, 1.009) 0.02
Hyperlipidemia							
Yes	316/5,057	1.01 (1.00, 1.02) 0.12	1.04 (1.02, 1.05) < 0.0001	1.02 (0.77, 1.36) 0.87	1.002 (1.001, 1.003) 0.001	1.50 (1.23, 1.83) 0.0001	1.005 (1.001, 1.008) 0.01
No	207/11,109	1.00 (0.99, 1.02) 0.46	1.04 (1.02, 1.06) < 0.0001	1.90 (1.31, 2.74) 0.0008	1.004 (1.002, 1.005) < 0.0001	1.89 (1.45,2.47) < 0.0001	1.007 (1.003,1.01) 0.0002

## Discussion

This cross-sectional study analyzed data from 16,092 participants who met the inclusion and exclusion criteria to explore the association between insulin resistance-related indices (HOMA-IR, METS-IR, TyG, TyG-WC, TyG-WHtR and TyG-BMI) and ASCVD with hyperuricemia. The results showed that after adjusting for covariates, individuals with higher METS-IR, TyG-WC, TyG-WHtR, and TyG-BMI values exhibited significantly higher risks of ASCVD with hyperuricemia. The RCS analysis confirmed nonlinear associations for these indices. Among these indices, TyG-WHtR exhibited the best performance in predicting CHD combined with hyperuricemia, heart attack combined with hyperuricemia, and stroke combined with hyperuricemia, demonstrating its unique superiority. The ROC curve further reinforced the strong predictive capabilities of METS-IR, TyG-WC, TyG-WHtR, and TyG-BMI, with AUC values greater than 0.85.

Previous studies have confirmed the association between IR and ASCVD, as well as the predictive and diagnostic capabilities of various IR-related indicators for ASCVD. Building on this foundation, our study focuses on the specific population of ASCVD combined with hyperuricemia, aiming to further explore the predictive ability of different IR indicators for this comorbidity. As early as 1996, a large cohort study found that higher insulin sensitivity was associated with a lower incidence of ASCVD in Hispanics and non-Hispanic whites, but not in blacks (12). Subsequent studies have further confirmed that insulin resistance is significantly associated with an increased risk of ASCVD and serves as an independent predictor of ASCVD (21–24). In the studies by Bonora et al. and Hedblad et al., insulin resistance was estimated using the HOMA method (22–24). IR has been established as an important contributor to the pathogenesis of ASCVD (25). In states of insulin resistance, impaired insulin signaling in endothelial cells, along with elevated levels of insulin and aldosterone, lead to reduced bioavailability of nitric oxide and vascular stiffness, thereby promoting the onset and progression of ASCVD (26).

Clinically, insulin resistance is associated with hyperuricemia. As uric acid levels increase, the prevalence of metabolic syndrome significantly rises, often presenting as comorbidities (27, 28). In addition, the study by Zhu et al. showed that as the level of hyperuricemia increases, the prevalence of comorbidities such as hypertension, obesity, heart failure, diabetes, myocardial infarction, and stroke rises to varying degrees (27). Meta-analyses have shown that serum uric acid levels are positively associated with the incidence of impaired fasting glucose and type 2 diabetes (29–31). Uric acid promotes insulin resistance by inhibiting the bioavailability of nitric oxide. In turn, high insulin levels resulting from insulin resistance suppress renal uric acid secretion and increase UA-producing substrates, leading to hyperuricemia (29). Uric acid also triggers inflammatory responses and alters the oxidative state of adipocytes, thereby contributing to various metabolic abnormalities, including insulin resistance (32).

There is an inherent close association between ASCVD and hyperuricemia. Increased serum uric acid levels are implicated in the pathogenesis of cardiovascular diseases (33). Previous meta-analyses have demonstrated a positive association between hyperuricemia and the incidence and mortality of CHD (34–36), the occurrence of heart failure (37), and an increased risk of stroke (38, 39). Research evidence suggests that hyperuricemia promotes the development of ASCVD through mechanisms such as inducing inflammation, endothelial dysfunction, vascular smooth muscle cell proliferation, and activation of the renin-angiotensin system (40). There is both pathological and epidemiological evidence indicating that high uric acid levels are closely associated with lifestyle factors, particularly alcohol consumption, physical activity, and smoking, as well as various metabolic indicators, including hypertension, high BMI, hyperglycemia, high triglycerides, and low HDL-C (41, 42). As a result, ASCVD is often accompanied by hyperuricemia.

Our study explored and compared HOMA-IR, METS-IR, TyG, and TyG-related indices in relation to ASCVD with hyperuricemia. Since the development of HOMA-IR in 1985, it has been widely utilized for the assessment of insulin resistance (43). HOMA-IR is the most commonly used insulin resistance index, but TyG index has been suggested as a reliable alternative insulin resistance index (43, 44). METS-IR also demonstrated strong performance in predicting ASCVD with hyperuricemia. Previous studies have shown that METS-IR is a strong predictor of cardiovascular disease, and is significantly associated with both all-cause mortality and cardiovascular mortality, outperforming HOMA-IR in predictive ability (45, 46). Moreover, the risk of cardiovascular disease (CVD) increases with the prolonged accumulation of METS-IR over time (47). This may be because the calculation of METS-IR requires not only fasting glucose and triglycerides but also BMI and HDL-C. Additionally, METS-IR is associated with fat accumulation in the liver, which is considered closely linked to the development of insulin resistance and cardiometabolic diseases (48). Notably, both the HOMA-IR and TyG exhibit a degree of “saturation” in their association with the occurrence of ASCVD combined with hyperuricemia. In contrast, METS-IR demonstrates a parabolic upward trend, particularly pronounced in the highest quartile. This pattern may be attributable to the inclusion of logarithmically transformed HDL-C in the METS-IR calculation. A retrospective cohort study involving 15,388 participants in Japan revealed a nonlinear relationship between HDL-C levels and diabetes risk, with extremely high HDL-C levels being associated with an increased risk of insulin resistance (49). Therefore, the use of log-transformed HDL-C in METS-IR may introduce a non-uniform distribution in its assessment of insulin resistance. HDL-C may exhibit compensatory elevation in the early stages of disease, while more pronounced changes are observed as the disease progresses.

Recent findings from the LoDoCo 1 and 2 trials, as well as the COLCOT study, have emphasized the residual cardiovascular risk that remains even with effective lipid control, underscoring the importance of factors beyond lipid levels in ASCVD risk (50–52). These studies highlight that inflammation and other metabolic abnormalities continue to contribute significantly to cardiovascular outcomes, even when lipid levels are managed. Although lipid control may not capture all residual cardiovascular risk, lipid-lowering therapy remains a cornerstone of ASCVD management, supported by extensive clinical evidence. Furthermore, contemporary research highlights the interaction between lipid metabolism, insulin resistance, and other metabolic abnormalities, suggesting these factors act synergistically in driving cardiovascular risk. METS-IR captures the complex interplay between these metabolic factors. This composite nature may provide a more comprehensive reflection of cardiovascular risk, particularly in individuals with advanced cardiometabolic disease.

In recent years, the TyG index has been confirmed as an effective indicator of insulin resistance (19, 20, 53). It is calculated using fasting triglycerides and glucose, making it simple and easily accessible. Multiple studies have previously indicated that the TyG index is significantly associated with cardiovascular disease risk and mortality (54–56). However, both of these indices did not demonstrate outstanding predictive abilities for ASCVD combined with hyperuricemia. Combining obesity-related indices, such as WC, WHtR, and BMI, with TyG significantly enhanced the association and predictive ability for ASCVD combined with hyperuricemia. In the aforementioned study by Zhu et al., it was mentioned that individuals with elevated uric acid levels have an increased risk of comorbid obesity and cardiovascular diseases, highlighting the association between obesity, hyperuricemia, and ASCVD (27). Studies have shown that combining TyG with obesity-related indices to form new markers, such as TyG-BMI or TyG-WC, provides better assessment and predictive performance compared to TyG alone (57, 58). The same results have been observed in ASCVD with hyperuricemia. In our study on ASCVD with hyperuricemia, TyG-WHtR had the highest OR, followed by TyG-WC, while TyG-BMI had the largest AUC. The underlying results and reasons are complex, and may be related to the associations between obesity itself and both ASCVD and hyperuricemia. Obesity, especially visceral fat accumulation, plays a role in the development of both conditions. TyG-WC and TyG-WHtR also exhibited a parabolic increasing trend similar to that of METS-IR, which is likely attributable to the incorporation of waist circumference in both indices, thereby accounting for central obesity. Obesity is closely associated with the onset, progression, and poor prognosis of cardiovascular disease (59, 60). A cross-sectional study has shown that TyG, TyG-WC, TyG-WHtR, and TyG-BMI are all clearly associated with CVD, with TyG-WHtR and TyG-WC demonstrating superior diagnostic performance compared to TyG (61). Wang et al.'s mediation analysis revealed that obesity is a key factor mediating the relationship between diet and hyperuricemia (62). The CARDIA study found that in both White and Black populations, BMI is positively associated with a 10-year change in uric acid levels, and WC was positively correlated with the 10-year change in uric acid in White individuals (63). A study in Korea found that in both sexes, higher BMI and WC, as well as obesity and central obesity, are high-risk factors for hyperuricemia (64). It is worth noting that TyG-WHtR demonstrated stronger associations with ASCVD with hyperuricemia, as well as with CHD, heart attack, and stroke combined with hyperuricemia. Compared to TyG-WC, which incorporates waist circumference, and TyG-BMI, which integrates weight and height, TyG-WHtR provides a more comprehensive representation of obesity type and fat distribution within the population. This calculation method is better suited to capturing the complex relationships between these factors and cardiovascular risk.

IR is closely associated with various metabolic diseases, including ASCVD and hyperuricemia. The incidence rates of ASCVD and hyperuricemia are rising globally, and their comorbid state not only exacerbates the health burden on patients but also increases the risk of cardiovascular events (65, 66). Existing studies have predominantly focused on the association between IR and individual diseases, whereas research on the mechanisms and epidemiological characteristics of IR in the context of ASCVD combined with hyperuricemia remains relatively limited. This study found that insulin resistance-related indices, particularly TyG-WHtR, TyG-WC, TyG-BMI, and METS-IR, demonstrated significant advantages in predicting the risk of ASCVD with hyperuricemia. These indices offer a straightforward and cost-effective approach to risk evaluation, making them especially suitable for implementation in resource-constrained healthcare systems. The exceptional performance of TyG-WHtR highlights its potentional as an ideal tool for identifying high-risk populations for ASCVD combined with hyperuricemia, particularly for the early detection of metabolic disturbances before clinical symptoms manifest. The integration of these indices into routine clinical practice could enhance chronic disease management by enabling early risk stratification, improving patient outcomes through timely intervention, and optimizing follow-up care. This study also explores the variability of IR across different populations (e.g., by gender, age, and metabolic status subgroups) to offer a foundation for personalized diagnosis and treatment. By leveraging easily accessible IR indicators, the study seeks to develop screening tools for high-risk populations, improving the early diagnosis rate of these conditions. Furthermore, by identifying the critical role of IR in disease onset, the study aims to promote the development of precision therapeutics and lifestyle interventions targeting IR mechanisms. Additional studies are needed to confirm the robustness of these indices across diverse populations and healthcare settings. Furthermore, evaluating their cost-effectiveness in large-scale screening programs will be crucial to justify their widespread adoption and ensure that they contribute meaningfully to reducing the global burden of ASCVD and metabolic disorders.

## Strengths and limitations

The strengths of this study are as follows: firstly, it focuses on the population with ASCVD combined with hyperuricemia, offering an innovative perspective on the interplay between insulin resistance and this comorbidity. Secondly, it evaluates and compares various insulin resistance-related indices, incorporating obesity-related indices with TyG. Thirdly, the study rigorously adjusts for multiple covariates, encompassing not only demographic factors but also clinical conditions such as hypertension, diabetes, and hyperlipidemia, thereby ensuring robust and reliable findings.

This study also has its limitations: The reliance on self-reported data for ASCVD diagnosis may have introduced recall bias and led to underdiagnosis of asymptomatic or undiagnosed cases. And participants on uric acid-lowering therapy were included, which may introduce heterogeneity in serum uric acid levels due to a small number of treated individuals. Additionally, the use of single-sample data for calculating insulin resistance-related indices captures only a transient state, potentially limiting the accuracy of long-term risk assessment. Lastly, the geographically restricted population sample may reduce the generalizability of the findings, necessitating further studies across diverse regions to validate the results.

## Conclusion

This NHANES-based study highlighted significant associations between insulin resistance indices, particularly METS-IR, TyG-WC, and TyG-WHtR, and ASCVD with hyperuricemia. Furthermore, it demonstrated the strong predictive capabilities of these indices for identifying individuals at risk for this comorbidity. These findings offer valuable insights into early detection and preventive strategies for ASCVD combined with hyperuricemia, emphasizing the practicality of these indices in clinical and public health settings. Future studies are expected to validate these findings in broader populations through large-scale cohort studies and randomized controlled trials (RCTs).

## Data Availability

The original contributions presented in the study are included in the article/Supplementary Material, further inquiries can be directed to the corresponding author.
